# Supplementary material for: From sequence to enzyme mechanism using multi-label machine learning
Source: BMC Bioinformatics. 2014 May 19;15:150. doi: 10.1186/1471-2105-15-150 (PMC4229970; doi:10.1186/1471-2105-15-150)
Supplement: Additional file 2 — Java code of ml2db. Additional file ml2db_code.tar.gz contains the Java source code to run the multi-label machine learning experiments and save the results to database. The code’s Javadoc is included. [file 1471-2105-15-150-S2.zip › additional file 2/ml2db/ml2db/doc/mulan/evaluation/measure/Measure.html]

Measure


JavaScript is disabled on your browser.


- Overview
- Package
- Class
- Use
- Tree
- Deprecated
- Index
- Help

- Prev Class
- Next Class

- Frames
- No Frames

- All Classes

- Summary:
- Nested |
- Field |
- Constr |
- Method

- Detail:
- Field |
- Constr |
- Method


mulan.evaluation.measure

## Interface Measure

- All Known Implementing Classes:
  :   AveragePrecision, BipartitionMeasureBase, ConfidenceMeasureBase, Coverage, ErrorSetSize, ExampleBasedAccuracy, ExampleBasedBipartitionMeasureBase, ExampleBasedFMeasure, ExampleBasedPrecision, ExampleBasedRecall, ExampleBasedSpecificity, GeometricMeanAverageInterpolatedPrecision, GeometricMeanAveragePrecision, HammingLoss, HierarchicalLoss, IsError, LabelBasedAUC, LabelBasedAveragePrecision, LabelBasedBipartitionMeasureBase, LabelBasedFMeasure, LabelBasedPrecision, LabelBasedRecall, LabelBasedSpecificity, LossBasedBipartitionMeasureBase, LossBasedRankingMeasureBase, MacroAUC, MacroFMeasure, MacroPrecision, MacroRecall, MacroSpecificity, MeanAverageInterpolatedPrecision, MeanAveragePrecision, MeasureBase, MicroAUC, MicroFMeasure, MicroPrecision, MicroRecall, MicroSpecificity, OneError, RankingLoss, RankingMeasureBase, SubsetAccuracy

  ---

    

  ```
  public interface Measure
  ```

  Interface for a measure, used to evaluate the performance of a multi-label learner
  on when performing multi-label learning task. Various measures capture different
  characteristics of a learning task performance.

  Author:
  :   Jozef Vilcek

- - ### Method Summary

    Methods

    | Modifier and Type | Method and Description |
    | --- | --- |
    | `double` | `getIdealValue()` Gets an 'ideal' value of a measure. |
    | `java.lang.String` | `getName()` Gets the name of a measure. |
    | `double` | `getValue()` Gets the value of a measure. |
    | `Measure` | `makeCopy()` Creates a deep copy of the given measure using serialization. |
    | `void` | `reset()` Resets the cumulated measure value, so the process of computation can be started from beginning (e.g. |
    | `void` | `update(MultiLabelOutput prediction, boolean[] truth)` Computes the value of a measure for the given prediction and true labels. |

- - ### Method Detail


    - #### getName

      ```
      java.lang.String getName()
      ```

      Gets the name of a measure.

      Returns:
      :   the name of a measure.


    - #### getValue

      ```
      double getValue()
      ```

      Gets the value of a measure. The measure is incrementally cumulated for learner's
      prediction by each `update(MultiLabelOutput, boolean[])` call. The value
      returned by the method, returns sum of all update calls divided by the number
      of calls (average of all measures for all predictions).

      Returns:
      :   the average measure value computed so far


    - #### getIdealValue

      ```
      double getIdealValue()
      ```

      Gets an 'ideal' value of a measure. The 'ideal' means, that the value
      represents the best achievable performance of a learner for an prediction of
      a multi-label task and associated true labels.

      Returns:
      :   the ideal value


    - #### update

      ```
      void update(MultiLabelOutput prediction,
                boolean[] truth)
      ```

      Computes the value of a measure for the given prediction and true labels. The immediate value of
      a measure is returned and result is added to the cumulated measure value.

      Parameters:
      :   `prediction` - the prediction for which measure has to be computed
      :   `truth` - the true labels bipartition for given prediction

      See Also:
      :   `getValue()`


    - #### makeCopy

      ```
      Measure makeCopy()
                       throws java.lang.Exception
      ```

      Creates a deep copy of the given measure using serialization.

      Returns:
      :   a deep copy of the measure

      Throws:
      :   `java.lang.Exception` - if an error occurs while making copy of the measure.


    - #### reset

      ```
      void reset()
      ```

      Resets the cumulated measure value, so the process of computation can be started
      from beginning (e.g. for a new series of outputs from learning task).


- Overview
- Package
- Class
- Use
- Tree
- Deprecated
- Index
- Help

- Prev Class
- Next Class

- Frames
- No Frames

- All Classes

- Summary:
- Nested |
- Field |
- Constr |
- Method

- Detail:
- Field |
- Constr |
- Method
